# First global report about the prevalence of multi-drug resistant *Haemophilus influenzae*: a systematic review and meta-analysis

**DOI:** 10.1186/s12879-023-08930-5

**Published:** 2024-01-15

**Authors:** Mohammad Abavisani, Masoud Keikha, Mohsen Karbalaei

**Affiliations:** 1 Student Research Committee, Mashhad University of Medical Sciences, Mashhad, Iran; 2 Department of Microbiology and Virology, School of Medicine, Iranshahr University of Medical Sciences, Iranshahr, Iran; 3 Department of Microbiology and Virology, School of Medicine, Jiroft University of Medical Sciences, Jiroft, Iran

**Keywords:** Beta-lactamase, Drug resistance, *Haemophilus influenzae*, Meta-analysis

## Abstract

**Background:**

In recent decades, the prevalence of antibiotic resistance is increasing in *Haemophilus influenzae* (*Haemophilus influenzae*), which poses important challenges to global health. This research offers a comprehensive meta-analysis of the global epidemiology of multi-drug resistant (MDR) *H. influenzae*.

**Methods:**

In this study, we conducted a meta-analysis based on PRISMA checklist. Electronic databases including PubMed, ISI Web of Science, Scopus, EMBASE, and Google Scholar were reviewed using keywords related to *H. influenzae* and antibiotic resistance. Eligible studies were selected based on stringent inclusion and exclusion criteria. Then, data from these studies were analyzed using the Comprehensive Meta-Analysis (CMA) software.

**Results:**

Of 375 retrieved articles, 16 met the inclusion criteria. These studies were conducted from 2003 to 2023 and analyzed data from 19,787 clinical isolates of *H. influenzae*. The results showed different levels of resistance of *H. influenzae* to different antibiotics: ampicillin (36%), azithromycin (15.3%), ceftriaxone (1.4%), etc. The global prevalence for beta-lactamases producing *H. influenzae* and MDR *H. influenzae* was measured 34.9% and 23.1%, respectively. The prevalence rate of MDR *H. influenzae* was higher in Asian countries (24.6%) compared to Western regions (15.7%). MDR *H. influenzae* had the highest prevalence in meningitis cases (46.9%) and the lowest prevalence in acute otitis media (0.5%).

**Conclusions:**

The prevalence of MDR *H. influenzae* has been increasing worldwide, especially in Asian regions. This highlights the urgent need for monitoring and implementation of effective antibiotic stewardship programs globally.

**Supplementary Information:**

The online version contains supplementary material available at 10.1186/s12879-023-08930-5.

## Background

The increase of antibiotic resistance in the era of modern medicine represents one of the most important challenges that the global health community is facing. At the center of this problem is the *Haemophilus influenzae* (*H. influenzae*) bacterium, which historically has been the leading cause of bacterial meningitis and other invasive conditions in pediatrics [1]. This Gram-negative coccobacillus presents with a variety of pathologies, causing conditions ranging from relatively benign otitis media to severe diseases such as septicemia [2]. Before to the advent of the *H. influenzae* type b (Hib) vaccine, the global burden of invasive Hib diseases was notably significant. Although this vaccine is advantageous in reducing the challenges caused by Hib, the apparent emergence of non-typeable *H. influenzae* (NTHi) strains has been implicated, particularly in respiratory pathologies [3, 4].

The presence of many challenges can lead to the acceleration of antibiotic resistance of this bacterium. The velocity and extent of antibiotic resistance, especially in bacterial species like *H. influenzae* are disconcertingly high [1, 5]. This intensification of resistance can be attributed to a combination of factors including, excessive use of antibiotics, self-administration of drugs, short treatment courses, and unrestricted antibiotic procurement in certain areas [6–8]. The implications of these actions are profound; the ineffectiveness of monotherapy leads to prolonged disease duration, increased health care costs, and increased mortality [9].

According to the registered reports, the phenomenon of antibiotic resistance in *H. influenzae* contains various pharmaceutical agents, from traditional drugs such as ampicillin and chloramphenicol to newer compounds like fluoroquinolones [10, 11]. The genetic basis of such resistance lies mainly in the absorption of resistance-causing genetic elements, which is facilitated through mechanisms such as conjugation and transformation. This evolving genetic perspective poses significant challenges to existing treatment strategies for clinicians, and complicates the treatment path for what was once a simple bacterial infection [12].

In the present meta-analysis we conducted a comprehensive study on the global antibiotic resistance of this bacterium, based on geographical distribution. We focused on MDR *H. influenzae* strains, which in turn highlights new antibiotic stewardship strategies against this pathogen in healthcare settings.

## Methods

### Search strategy

In the present study, we conducted a comprehensive systematic review and meta-analysis on the prevalence of multidrug-resistant *H. influenzae* worldwide, using the Preferred Reporting Items for Systematic Reviews and Meta-Analyses (PRISMA) checklist [13]. Major electronic databases, namely Medline, ISI Web of Science, Scopus, EMBASE, Google Scholar, and ProQuest were scoured. Aligned with the Medical Subject Headings (MeSH), Key terms such as “*Haemophilus influenzae*”, “*H. influenzae*”, “Antibiotic resistance”, “Multi-drug resistance”, and “MDR”, were integrated in this research. The search had no restrictions on language or date of publication. To ensure completeness, article citations were manually checked to identify any potentially overlooked studies.

### Study selection according to the Clinical and Laboratory Standards Institute

In order to assess the eligibility of documents, all content of articles including title, abstract, and full text of relevant studies were evaluated. Inclusion criteria were: 1) original studies that investigated the prevalence of MDR *H. influenzae* in clinical samples; 2) articles related to *H. influenzae* infection in human subjects; 3) retrospective as well as cross-sectional studies; 4) articles that evaluated antimicrobial/antibiotic susceptibility testing (AST) according to the Clinical and Laboratory Standards Institute (CLSI) guideline. Our exclusion criteria were as follows: 1) duplicate studies; 2) article types (e.g. letters to the editor, case reports, reviews, and congress abstracts); 3) animal studies; 4) studies with insufficient information. Two independent authors participated in this step and discrepancies were resolved through discussion.

### Quality appraisal and data extraction

The Joanna Briggs Institute (JBI) checklist was used to assess the quality assessment of relevant studies [14]. In this context, studies were included if they achieved at least 6 scores. Next, the required information was extracted from eligible studies, including: I) first author, II) publication year, III) country, IV) infection type, V) number of *H. influenzae* isolates, VI) prevalence of antibiotic resistance to ampicillin, amoxicillin, tetracycline, chloramphenicol, cefotaxime, ciprofloxacin, rifampin, sulfamethoxazole, cefuroxime, azithromycin, cefotaxime, ceftriaxone, levofloxacin, meropenem, VII) prevalence of beta-lactamase strains, VIII) prevalence of MDR *H*. *influenzae*, as well as IX) diagnostic method. Two independent authors were involved in the process, and discordance was determined by a third author.

### Statistical analysis

Data were synthesized using the Comprehensive Meta-Analysis (CMA) software, version 2.2 (Biostat, Englewood, NJ). The Cochrane Q-test (*p* < 0.05) and the I-squared (*I*^*2*^) index were used to measure the heterogeneity of studies. In case of significant heterogeneity, random-effects model based on the DerSimonian and Laird approach was applied. In addition, meta-regression techniques were used to investigate the impact of potential moderators. Publication bias was evaluated through the Egger’s *p* value test, Begg’s *p* value test, and funnel plot. If significant publication bias was detected, the trim-fill method was used to estimate any potential missing studies.

## Results

### Literature search

**Fig. 1:**
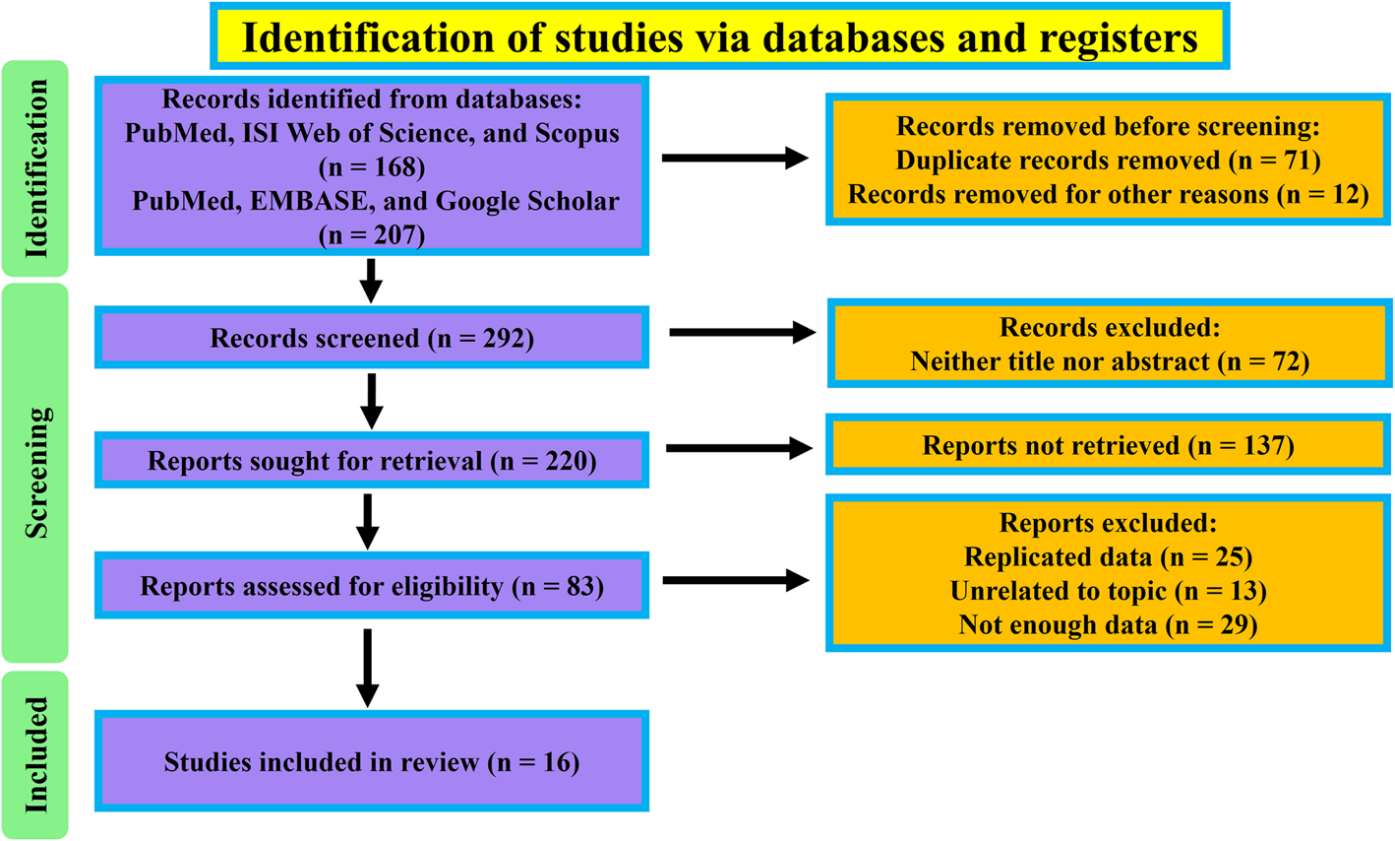
Flow-diagram of study selection process

Overall, 375 pertinent documents were retrieved from database searches (Fig. 1). After initial evaluation of titles and abstracts, 209 articles were excluded. The main reasons for removing duplicates were included: non-original researches, animal-based studies, and the absence of reports on MDR *H. influenzae*. A comprehensive evaluation of the full text of 83 papers was then performed for potential inclusion. Upon further scrutiny, and supplemented by manual bibliographic searches, a total of 16 studies met the criteria for inclusion in our systematic review and meta-analysis. The data of these studies are summarized in Table 1 [1, 10, 11, 15–27].

**Table 1:** Characteristics of included studies

First author	Year	Country	Infection type	Number of *H. influenzae*			Antibiotic Resistance (%)															Diagnostic method	Ref
				Total	serotype b	Non-b-Type	AMP	AMOX	SXT	CFX	CEF	CFO	LEV	MER	TET	CIP	CHL	AZT	RIF	Bla	MDR		
Tamargo	2003	Spain	Meningitis	938	938	0	46.3	NR	51.3	NR	0	NR	NR	NR	33.2	NR	44.0	NR	0	46.3	43.8	BM	[19]
Campos	2003	Spain	Invasive	49	0	49	24.5	NR	44.9	NR	NR	NR	NR	NR	12.2	NR	14.3	NR	2	NR	18.4	MIC	[20]
Bajanca	2004	Portugal	Invasive	119	72	47	24.4	0	NR	NR	0	NR	NR	NR	12.6	0	10.1	NR	0	26.9	34.4	NR	[21]
Shen	2004	China	Invasive	898	NR	NR	10.6	0	35	0	35	35	35	35	35	1	35	35	35	12	12.8	E-test	[27]
Shen	2007	China	ARI	798	NR	NR	12	0	29.8	0	0	NR	NR	NR	NR	1	NR	0	NR	12.0	12.8	DD	[15]
Wang	2008	Taiwan	ARI	26	12	14	60.9	NR	NR	NR	NR	NR	NR	NR	NR	NR	NR	NR	NR	61.5	26.9	NR	[26]
Rahman	2008	Bangladesh	Invasive	73	59	14	31	0	44	22	NR	NR	0	NR	NR	0	42	1.4	NR	NR	16	MIC	[18]
Mojgani	2011	Iran	Invasive	38	13	35	43.6	NR	47.1	NR	NR	NR	NR	NR	NR	NR	NR	NR	NR	NR	18.42	NR	[25]
Pumarola	2013	Spain	acute otitis media	54	NR	NR	15	13	NR	NR	NR	NR	NR	NR	NR	NR	NR	NR	NR	11	0	DD	[17]
Chen	2018	China	ARI	402	NR	NR	61.4	NR	64.4	28.4	NR	11.2	NR	5.2	NR	2.7	NR	17.4	NR	42.0	15.2	E-test	[11]
Yamada	2019	Japan	Invasive	260	0	260	NR	NR	NR	NR	NR	NR	1.15	NR	NR	NR	NR	NR	NR	26.53	0.38	MIC	[10]
Awulachew	2020	Ethiopia	Meningitis	4	4	0	50	NR	25	NR	25	NR	NR	NR	75	25	25	NR	50	NR	50	DD	[16]
Su	2020	Taiwan	Invasive	2091	NR	NR	54.97	16.45	54.85	7.6	NR	NR	14.1	NR	NR	NR	20.9	NR	NR	50.37	26.6	DD	[1]
Zhou	2021	China	Invasive	13,810	NR	NR	69.37	NR	70.98	51.35	2.68	0.16	NR	0.95	NR	NR	2.99	38.21	NR	63.32	84.94	DD	[22]
Derrick	2023	Australia	ARI	79	0	79	6.3	NR	10.1	NR	0	NR	NR	0	0	0	0	NR	NR	NR	1.72	MIC	[24]
Yuan	2023	China	ARI	148	NR	NR	73.37	29	78.87	41.87	NR	NR	NR	NR	7.5	NR	5.62	NR	NR	59.5	62.23	MIC	[23]

*Abbreviations:* *AMP* Ampicillin, *TET* Tetracycline, *CHL* Chloramphenicol, *AMOX* Amoxicillin, *CEF* Cefotaxime, *CIP* Ciprofloxacin, *RIF* Rifampin, *SXT* Sulfamethoxazole, *CFX* Cefuroxime, *AZT* Azithromycin, *CFO* Cefotaxime, *CEF* Ceftriaxone, *LEV* Levofloxacin, *MER* Meropenem, *Bla* Beta-lactamase, *BM* Broth microdilution, *ARI* Acute Respiratory Infections, *DD* Disc diffusion

### Characteristics of included studies

These investigations focused on the prevalence of MDR *H. influenzae* in different regions: Spain (*n* = 3), Portugal (*n* = 1), China (*n* = 5), Taiwan (*n* = 2), Bangladesh (*n* = 1), Iran (*n* = 1), Japan (*n* = 1), Ethiopia (*n* = 1), and Australia (*n* = 1). The time range of these studies covers from 2003 to 2023. The methods used in the studies included the evaluation of antibiotic susceptibility of clinical isolates of *H. influenzae*; for example disc diffusion, E-test, and broth dilution techniques. These clinical species of *H. influenzae* were isolated from a wide range of disorders including invasive infections, meningitis, acute otitis media (AOM), and acute respiratory infections. Cumulatively, in our analysis, we incorporated data from 19,787 *H. influenza*e clinical isolates, which encompassed both Hib and non-Hib serotypes. It is noteworthy that two studies exclusively assessed the AST of the Hib serotype infections [16, 19]. Of the total *H. influenzae* isolates, approximately 46.8 ± 8.9% were identified as Hib strain.

### Characteristics of *H. influenzae* antibiotic resistance

In this meta-analysis, the antibiotic resistance trends of *H. influenzae* were determined as follows: amoxicillin, 6.3% (95% CI: 2.5–15; *I*^*2*^: 84.33; *p* = 0.01; Egger’s *p* = 0.01; Begg’s *p* = 0.02), ampicillin, 36% (95% CI: 25.6–48; *I*^*2*^: 94.38; *p* = 0.01; Egger’s *p* = 0.01; Begg’s *p* = 0.01), azithromycin, 15.3% (95% CI: 6.7–31.1; *I*^*2*^: 89.28; *p* = 0.01; Egger’s *p* = 0.03; Begg’s *p* = 0.05), ceftriaxone, 1.4% (95% CI: 0.2–10.2; *I*^*2*^: 73.91; *p* = 0.01; Egger’s *p* = 0.1; Begg’s *p* = 0.4), cefotaxime, 3.6% (95% CI: 1.3–9.5; *I*^*2*^: 80.17; *p* = 0.01; Egger’s *p* = 0.03; Begg’s *p* = 0.1), cefuroxime, 19.1% (95% CI: 9.7–34.0; *I*^*2*^: 91.14; *p* = 0.01; Egger’s *p* = 0.03; Begg’s *p* = 0.01), chloramphenicol, 17.2% (95% CI: 10.3–27.1; *I*^*2*^: 90.59; *p* = 0.01; Egger’s *p* = 0.1; Begg’s *p* = 0.01), ciprofloxacin, 1.7% (95% CI: 0.3–8.8; *I*^*2*^: 88.27; *p* = 0.01; Egger’s *p* = 0.01; Begg’s *p* = 0.5), levofloxacin, 7.5% (95% CI: 1.9–25.5; *I*^*2*^: 90.78; *p* = 0.01; Egger’s *p* = 0.09; Begg’s *p* = 0.3), meropenem, 4.3% (95% CI: 0.6–26.0; *I*^*2*^: 92.77; *p* = 0.01; Egger’s *p* = 0.07; Begg’s *p* = 0.5), rifampin, 8.9% (95% CI: 2.5–27.2; *I*^*2*^: 92.31; *p* = 0.01; Egger’s *p* = 0.01; Begg’s *p* = 0.3), sulfamethoxazole, 45.6% (95% CI: 34.9–56.7; *I*^*2*^: 92.39; *p* = 0.01; Egger’s *p* = 0.2; Begg’s *p* = 0.3), and tetracycline, 19.9% (95% CI: 8.3–40.4; *I*^*2*^: 95.3; *p* = 0.01; Egger’s *p* = 0.08; Begg’s *p* = 0.1).

### Characteristics of MDR *H. influenzae*

**Fig. 2:**
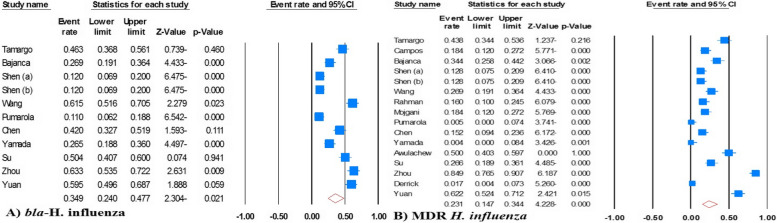
Forest plot of meta-analysis on the prevalence of beta-lactamases producing *H. influenzae* and multidrug resistant *H. influenzae*

The global prevalence of beta-lactamases producing *H. influenzae* and MDR *H. influenzae* was established at 34.9% (95% CI: 24.0-47.7; *I*^*2*^: 93.56; *p* = 0.01; Egger’s *p* = 0.01; Begg’s *p* = 0.01) and 23.1% (95% CI: 14.7–34.4; *I*^*2*^: 93.9; *p* = 0.01; Egger’s *p* = 0.04; Begg’s *p* = 0.01), respectively (Fig. 2). Furthermore, our data revealed an increasing trend in the prevalence of beta-lactamases producing *H. influenzae*, from 22.1% (95% CI: 10.4–40.9) during 2003–2007 to 48.1% (95% CI: 35.5–61.0), a more than twofold increase, during 2019–2023. In contrast, the trend for MDR *H. influenzae* has remained stable over the past two decades. Specifically, the pooled prevalence rates of MDR *H. influenzae* were 22.8% (95% CI: 13.0-36.8), 20.8% (95% CI: 16.5–25.9), and 27.8% (95% CI: 11.8–52.5), during 2003–2007, 2008–2012, and 2019–2023, respectively.

After analyzing the geographical distribution, it was found that the prevalence of MDR *H. influenzae* in Asian countries was significantly higher than in Western regions, with rates of 24.6% (95% CI: 12.9–41.8) and 15.7% (95% CI: 6.7–32.6), respectively. When the study was classified according to the type of infection, the incidence of MDR *H. influenzae* was most pronounced in cases of meningitis with 46.9% (95% CI: 40.1–53.9) and the lowest prevalence was related to cases of AOM with 0.5% (95% CI: 0.0-7.4). The overall prevalence of MDR *H. influenzae* for invasive infections was 24.1% (95% CI: 12.0-42.5), while for acute respiratory infections it was 18.2% (95% CI: 6.6–41.1).

In addition, a meta-regression analysis was performed to examine the potential effects of several moderating factors including, publication year, methodology, geographical latitude, and type of infection, on the pooled estimates. The results showed that the year of publication had a discernible impact on the aggregated estimates pertaining to MDR *H. influenzae* infection, as described in Table 2.

**Table 2:** Meta-regression analysis of moderator variables influencing the overall estimates regarding prevalence of MDR H. influenzae

Moderators	Coefficient	SE	95% CI	p-value
Year of publication	0.36	0.05	0.26–0.46	0.01
Method	0.08	0.11	0.31 − 0.14	0.46
Latitude	0.79	0.26	1.30 − 0.27	0.86
Infection type	0.31	0.25	0.81 − 0.17	0.20

### Publication bias

**Fig. 3:**
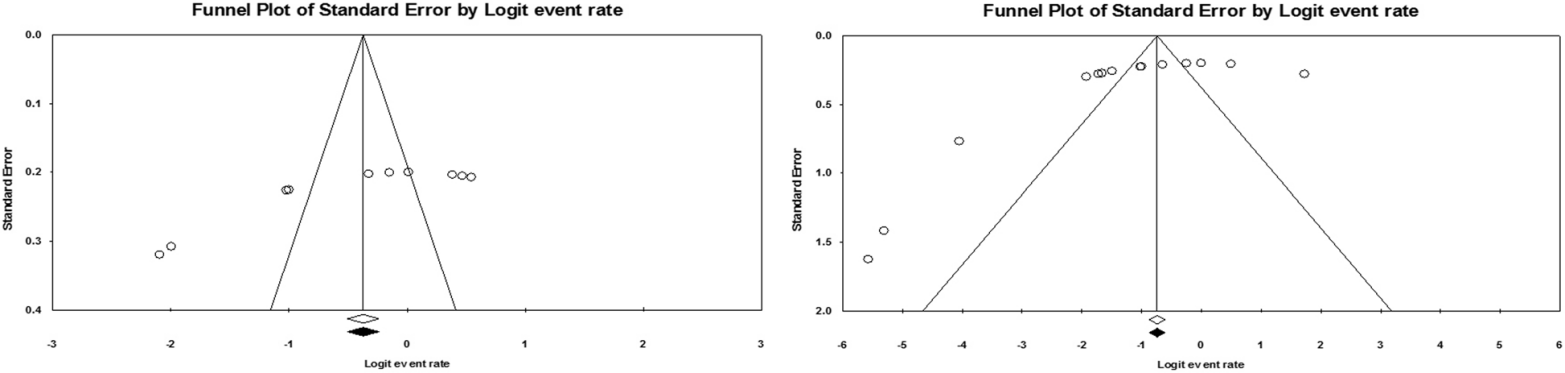
Funnel plot depicting publication bias of studies reporting the prevalence MDR *H. influenzae*

In the present meta-analysis, potential publication bias was carefully assessed using both Begg’s and Egger’s *p* value tests. Moreover, any observed asymmetry in the funnel plot was interpreted as indicating significant publication bias. Collective evidence from these methods confirms the presence of publication bias in the studies included in this analysis. Notwithstanding this, the trim-fill method was used to rectify and stabilize the overall effect estimates. The results after applying the trim-fill method further bolstered the strength of the pooled estimates (Fig. 3).

## Discussion

Our meta-analysis sheds light on the complex global patterns of MDR *H. influenzae* outbreaks. To the best of our knowledge, this is the first global meta-analysis on the prevalence of MDR *H. influenzae*. One of the notable observations is the high prevalence of this bacterium in Asian countries (24.6%) compared to Western regions (15.7%). This notable difference can be caused by both internal and external factors in the healthcare system. Previous research on the *H. influenzae* infection in Taiwan identified the presence of extensively drug-resistant (XDR) *H. influenzae* strains as early as 2007, and documented the consistent drug resistance status maintained by these strains [1]. Also, another study that focused on antibiotic susceptibility in Africa from 1978 to 2011 was consistent with our findings; emphasizing the non-susceptibility of *H. influenzae* isolates to several useful antibiotics; according to their statistics, the rate of non-susceptibility to erythromycin, trimethoprim/sulfamethoxazole, tetracycline, ampicillin (or penicillin) was measured as 69.8%, 48.1%, 37.5%, and 34.7%, respectively [28]. In addition, the results of resistance to several antibiotics in Africa such as ampicillin (34.7%), ceftriaxone (0.9%), cefotaxime (2.6%), and trimethoprim/sulfamethoxazole (48.1%) were almost similar to our results in the present meta-analysis. The number of isolates and the type of geographical region are considered as the two main reasons for the difference between the studies.

Some antibiotic-resistant bacteria such as sulfonamide-resistant *Streptococcus pyogenes* and penicillin-resistant *Staphylococcus aureus*, primarily have been linked to hospitals, places where we are faced with high consumption of antibiotics [29]. Such environments may serve as centers for the development and spread of drug resistance. Several factors, such as antibiotic prescribing habits, access to health care, and population density, all influence resistance patterns [30]. Notably, there is a significant variation in the prevalence of MDR *H. influenzae* among different infections. To illustrate, the occurrence of meningitis at 46.9% in contrast to AOM at 0.5% may be attributed to changes in bacterial pathogenicity, antibiotic utilization, laboratory diagnostic techniques, and host response. In essence, these factors collectively contribute to the observed difference in the incidence of meningitis and AOM [31].

Adding to the problem is that certain regions such as Asia and Central/Southern Europe have reported significantly lower incidence rates compared to other global regions. Interestingly, a stepwise logistic regression analysis from a study on 2091 *H. influenzae* isolates with disc diffusion-based AST elucidated specific demographic patterns, showing that male patients were less likely to harbor MDR *H. influenzae* strains [1]. Despite our focus on MDR *H. influenzae*, in many cases, antimicrobial prescriptions are made without knowledge of the causative organism. Vancomycin plus cefotaxime or ceftriaxone is the standard empirical antibiotic therapy for bacterial meningitis in children and newborns. On the other hand, azithromycin and clarithromycin are also alternative treatments in patients with AOM who have penicillin allergy [12, 32].

In our study, the prevalence of resistant *H. influenzae* to azithromycin was 15.3%. Given our initial concern about multi-drug resistance in *H. influenzae*, it is important to monitor changes in resistance patterns against a broader range of antibiotics. While the prevalence of invasive infections (24.1%) and acute respiratory infections (18.2%) is at steady state, it indicates a uniform degree of antibiotic resistance spread in these categories, which requires ‎equal attention. ‎After meningitis, childhood pneumonia and bacteremia are the most common diseases caused by Hib strains, and pneumonia is particularly dominant in developing countries [33]. The potential effect of seasonality on the prevalence of antimicrobial resistance is consistent. In a systematic review and meta-analysis by Martinez et al., they observed stable antimicrobial resistance rates in colder months for *S. pneumoniae* [34].

This observation aligns with our findings and suggest that respiratory infections, which are more common in colder seasons, maintain a consistent antibiotic resistance. In our meta-analysis, the global prevalence of beta-lactamases producing *H. influenzae* was established at 34.9%, which should be considered as a serious concern. This enzyme confers resistance against a variety of penicillin-based drugs by hydrolyzing their beta-lactam ring structure. Our findings are in line with a systematic review and meta-analysis operated by Mather et al., where resistance in Gram-negative bacteria, including *H. influenzae*, was frequently reported in terms of their ability to produce beta-lactamase enzymes [35]. Also, a particularly alarming observation from another study by Ginsburg et al. is the increasing trend of beta-lactamase production among Hib isolates, indicating a new concern for the African continent [28].

In addition, we showed that the prevalence of beta-lactamase producing *H. influenzae* is increasing, from 22.1% in 2007 − 2003 to 48.1% in 2023 − 2019. This surge emphasizes the heightened clinical reliance on beta-lactamases for treating *H. ‎influenzae*-mediated infections. Ceftriaxone, cefotaxime, or cefuroxime are also suggested for the treatment of pneumonia and bacteremia caused by beta-lactamase producing *H. influenzae* strains; on the other hand, ampicillin is suggested for beta-lactamase-negative strains [36]. Based on our results, the prevalence of resistance to ceftriaxone, cefotaxime, cefuroxime, and ampicillin was 1.4%, 4.1%, 19.1%, and 36%, respectively. In another meta-analysis conducted by Vaez et al., the prevalence of *H. influenzae* strains resistant to these antibiotics was estimated at 33.1%, 22.3%, 13.7%, and 54.8%, respectively [37].

Despite the modest increase in the pooled prevalence of both beta-lactamase producing *H. ‎influenzae* and MDR *H. influenzae*, it is clear that we stand on precarious ground.‎ It seems necessary to formulate and implement an antibiotic stewardship strategy to neutralize the emergence of XDR *H. influenzae* strains. Although the present study is comprehensive, it is not without limitations; the observed publication ‎bias, despite adjustments, may still affect the final pooled estimates. Furthermore, meta-analysis relies on published data, which may not represent unpublished studies or gray literature, potentially leading to over- or underestimation of true prevalence. In addition, inherent changes in the included studies in terms of methodology, sample size, and demographic distribution can cause heterogeneity in the results.

On the other hand, the strengths of this study lie in its expansive scope, detailed methodological approach, incorporation of a broad range of geographies, and infection types. We believe this study can offers a robust overview of the global landscape of MDR *H. influenzae*, serving as a pivotal resource for clinicians, researchers, and policymakers. Finally, while this meta-analysis offers pivotal insights into the prevalence of MDR *H. influenzae* across geographies and infection types, continued vigilance and updated research are essential to track, understand, and mitigate the spread of antibiotic resistance globally.

## Conclusions

The global health community is facing a daunting challenge in the field of antibiotic resistance, with *H. influenzae* at the forefront. Our comprehensive meta-analysis shows an alarming increase in resistance, especially for beta-lactamase producing strains, which almost doubled from 2003 to 2023. Although the rate of MDR *H. influenzae* has remained relatively stable over the past two decades, its continued prevalence is particularly concerning in cases of meningitis. According to our results, it seems that there is a higher prevalence of MDR *H. influenzae* in Asian countries as compared to the Western countries. In general, therapeutic measures include implementation of stewardship programs, appropriate use of antibiotics, public awareness campaigns, and conducting new treatment research.

### Supplementary Information

**Additional file 1.** Search strategy.

## Data Availability

All data generated or analyzed during this study are included in this published article.

## Abbreviations

*H. influenzae*: *Haemophilus influenzae*
MDR: Multidrug-resistant
MDR *H. influenzae*: Multidrug-resistant *H. influenzae*
PRISMA: Preferred Reporting Items for Systematic Reviews and Meta-Analyses
JBI: Joanna Briggs Institute
CMA: Comprehensive Meta-Analysis
CI: Confidence interval
OR: Odds ratio
