# Supplementary material for: First global report about the prevalence of multi-drug resistant Haemophilus influenzae: a systematic review and meta-analysis
Source: BMC Infect Dis. 2024 Jan 15;24:90. doi: 10.1186/s12879-023-08930-5 (PMC10789054; doi:10.1186/s12879-023-08930-5)
Supplement: Supplementary file 1 — Additional file 1. Search strategy. [file 12879_2023_8930_MOESM1_ESM.docx]

**Supplementary file: Search strategy**

**PubMed/MedLine**

“*Haemophilus influenzae*” OR “*H. influenzae*”; “*Haemophilus influenzae*” ‎[MeSH Terms]‎ AND “*H. influenzae*”; “Multi-drug resistance” OR “MDR”; “*H. influenzae*” ‎[MeSH Terms]‎ AND “Multi-drug resistance”; “Multi-drug resistant *Hemophilus influenzae*” ‎[MeSH Terms]‎ OR “MDR *H. influenzae*” ‎[MeSH Terms]‎; “*Haemophilus influenzae*” ‎[MeSH Terms]‎ AND “Multi-drug resistant *Hemophilus influenzae*” ‎[MeSH Terms]‎; “*H. influenzae*” ‎[MeSH Terms]‎ AND “MDR *H. influenzae*”; “Antibiotic resistance” ‎[MeSH Terms]‎ AND “MDR *H. influenzae*”; “Antibiotic resistance” ‎[MeSH Terms]‎ AND “Multi-drug resistant *Hemophilus influenzae*”

**EMBASE**

“*Haemophilus influenzae*” OR “*H. influenzae*”; “*Haemophilus influenzae*” AND “*H. influenzae*”; “Multi-drug resistance” OR “MDR”; “*H. influenzae*” AND “Multi-drug resistance”; “Multi-drug resistant *Hemophilus influenzae*” OR “MDR *H. influenzae*”‎; “*Haemophilus influenzae*” ‎AND “Multi-drug resistant *Hemophilus influenzae*”‎; “*H. influenzae*” AND “MDR *H. influenzae*”; “Antibiotic resistance”‎ AND “MDR *H. influenzae*”; “Antibiotic resistance” AND “Multi-drug resistant *Hemophilus influenzae*”

**WEB OF SCIENCE**

“*Haemophilus influenzae*” OR “*H. influenzae*”; “*Haemophilus influenzae*” AND “*H. influenzae*”; “Multi-drug resistance” OR “MDR”; “*H. influenzae*” AND “Multi-drug resistance”; “Multi-drug resistant *Hemophilus influenzae*” OR “MDR *H. influenzae*”‎; “*Haemophilus influenzae*” ‎AND “Multi-drug resistant *Hemophilus influenzae*”‎; “*H. influenzae*” AND “MDR *H. influenzae*”; “Antibiotic resistance”‎ AND “MDR *H. influenzae*”; “Antibiotic resistance” AND “Multi-drug resistant *Hemophilus influenzae*”

**SCOPUS**

“*Haemophilus influenzae*” OR “*H. influenzae*”; “*Haemophilus influenzae*” AND “*H. influenzae*”; “Multi-drug resistance” OR “MDR”; “*H. influenzae*” AND “Multi-drug resistance”; “Multi-drug resistant *Hemophilus influenzae*” OR “MDR *H. influenzae*”‎; “*Haemophilus influenzae*” ‎AND “Multi-drug resistant *Hemophilus influenzae*”‎; “*H. influenzae*” AND “MDR *H. influenzae*”; “Antibiotic resistance”‎ AND “MDR *H. influenzae*”; “Antibiotic resistance” AND “Multi-drug resistant *Hemophilus influenzae*”

**Google Scholar**

“*Haemophilus influenzae*” OR “*H. influenzae*”; “*Haemophilus influenzae*” AND “*H. influenzae*”; “Multi-drug resistance” OR “MDR”; “*H. influenzae*” AND “Multi-drug resistance”; “Multi-drug resistant *Hemophilus influenzae*” OR “MDR *H. influenzae*”‎; “*Haemophilus influenzae*” ‎AND “Multi-drug resistant *Hemophilus influenzae*”‎; “*H. influenzae*” AND “MDR *H. influenzae*”; “Antibiotic resistance”‎ AND “MDR *H. influenzae*”; “Antibiotic resistance” AND “Multi-drug resistant *Hemophilus influenzae*”

**ProQuest**

“*Haemophilus influenzae*” OR “*H. influenzae*”; “*Haemophilus influenzae*” AND “*H. influenzae*”; “Multi-drug resistance” OR “MDR”; “*H. influenzae*” AND “Multi-drug resistance”; “Multi-drug resistant *Hemophilus influenzae*” OR “MDR *H. influenzae*”‎; “*Haemophilus influenzae*” ‎AND “Multi-drug resistant *Hemophilus influenzae*”‎; “*H. influenzae*” AND “MDR *H. influenzae*”; “Antibiotic resistance”‎ AND “MDR *H. influenzae*”; “Antibiotic resistance” AND “Multi-drug resistant *Hemophilus influenzae*”
